# Treating Diabetic Foot Osteomyelitis: A Practical State-of-the-Art Update

**DOI:** 10.3390/medicina57040339

**Published:** 2021-04-01

**Authors:** Benjamin A. Lipsky, İlker Uçkay

**Affiliations:** 1 Department of Medicine, University of Washington, Seattle, WA 98116, USA; 2 Department of Orthopedic Surgery, Balgrist University Hospital, University of Zurich, 8008 Zurich, Switzerland; ilker.uckay@balgrist.ch

**Keywords:** diabetic foot osteomyelitis, antibiotic therapy, antibiotic stewardship, foot surgery, clinical outcomes, amputations, clinical research

## Abstract

*Background and Objectives:* Diabetic foot osteomyelitis (DFO) can be difficult to treat and securing optimal clinical outcomes requires a multidisciplinary approach involving a wide variety of medical, surgical and other health care professionals, as well as the patient. Results of studies conducted in the past few years have allowed experts to formulate guidelines that can improve clinical outcomes. *Material and Methods:* We conducted a narrative review of the literature on treat- ment of DFO, with an emphasis on studies published in the last two years, especially regarding antimicrobial therapies and surgical approached to treatment of DFO, supplemented by our own extensive clinical and research experience in this field. *Results:* Major amputations were once com- mon for DFO but, with improved diagnostic and surgical techniques, “conservative” surgery (foot- sparing, resecting only the infected and necrotic bone) is becoming commonplace, especially for forefoot infections. Traditional antibiotic therapy, which has been administered predominantly in- travenously and frequently for several months, can often be replaced by appropriately selected oral antibiotic regimens following only a brief (or even no) parenteral therapy, and given for no more than 6 weeks. Based on ongoing studies, the recommended duration of treatment may soon be even shorter, especially for cases in which a substantial portion of the infected bone has been resected. Using the results of cultures (preferably of bone specimens) and antimicrobial stewardship princi- ples allows clinicians to select evidence-based antibiotic regimens, often of a limited pathogen spec- trum. Intra-osseous antimicrobial and surgical approaches to treatment are also evolving in light of ongoing research. *Conclusions:* In this narrative, evidenced-based review, taking consideration of principles of antimicrobial stewardship and good surgical practice, we have highlighted the recent literature and offered practical, state-of-the-art advice on the antibiotic and surgical management of DFO.

## 1. Introduction

Infections of the foot are a frequent and serious problem in persons with diabetes mellitus. While these infections almost always begin in an open wound in the skin and soft tissue, they often spread contiguously to underlying bone. The resulting diabetic foot osteomyelitis (DFO) is thus usually a consequence of long-standing diabetes, most often related to advanced peripheral neuropathy, often coupled with peripheral arterial disease, foot deformities and suboptimal patient compliance with foot care recommendations [1,2,3,4]. The most serious and feared complication of DFO is lower extremity amputation. This outcome, the most common cause of which in developed countries is now the consequences of complications of diabetes, is associated with a five-year mortality rate of about 50% [5], which is higher than that for most cancers. Yet, in most cases this highly morbid outcome is preventable with optimal medical and surgical treatment. In this narrative review, based on a thorough search of the recent literature and our own extensive clinical and research experience, we highlight the current, practical state-of-the-art of the antibiotic and surgical therapies for DFO.

## 2. Materials & Methods

For this narrative (not systematic) review, we conducted an extensive literature search using PubMed and internet Google Scholar, with variations on the search terms “diabetic foot,” “osteomyelitis,” and “infection treatment”, seeking original research papers targeting antibiotic or surgical therapy of DFO, with a special emphasis on those published in the last two years. We only included papers written in English, and those reporting clinical research on human patients. We elected to exclude papers not focused on DFO, but aimed at exploring other aspects of diabetic foot infection (DFI), e.g., pathophysiology, biofilm, imaging, vasculopathy, histology, patient’s adherence, pressure off-loading, reconstructive surgery, or glycemic control, as they have been reviewed in other articles or guidelines [1,3,6,7,8]. In addition to the literature, the recommended approach to treating DFO that we offer is informed by our own clinical and research work in the field from 1985 to the present.

## 3. Results

### 3.1. Causative Pathogens

Any microorganism that is part of the resident or transient flora of the skin of the foot can colonize a break in the skin and cause an infection, which can then spread contiguously to underlying bone. The etiologic agents causing DFIs depend on many factors, including the geographic location of the patient (e.g., climate and socio-economic issues), the chronicity of the wound, where the infection was acquired (e.g., home versus an institution), and specific exposures (e.g., water borne pathogens like *Pseudomonas aeruginosa*). Most DFOs in North American and European countries are caused by aerobic gram-positive cocci, especially *Staphylococcus aureus*, and to a lesser extent ß-hemolytic *Streptococcus* species, enterococci [9], gram-negative bacilli [10], and coagulase-negative staphylococci. Wound cultures from a patient presenting in a warm climate, or with a chronic infection, or who has been recently treated with antimicrobials, are more likely to grow aerobic gram-negative organisms, including *Enterobacteriaceae* and *P. aeruginosa*. Obligately anaerobic bacteria are likely present in many infections (as shown by recent molecular microbiological studies), but are relatively infrequently isolated or reported by clinical microbiology laboratories. When anaerobes are reported as likely pathogens, they are most often isolated from infections with necrotic or ischemic areas [11].

Sometimes, bacteria causing DFO are resistant to commonly used antibiotics, especially if the patient is, or recently has been, receiving systemic antibiotic therapy or been in a health-care institution [12,13]. The most common drug-resistant pathogens in DFO are non-fermenting gram-negative rods, methicillin-resistant *S. aureus* (MRSA) [14] and methicillin-resistant coagulase-negative staphylococci (e.g., *S. epidermidis*). The incidence of MRSA as a pathogen in DFO, after rising in the late 20th and early 21st century, has (for unclear reasons) decreased more recently. Rates of antibiotic resistant pathogens vary widely, making it incumbent upon prescribing clinicians to keep updated on their prevalence in their own work site [1].

Several studies have shown that for a patient with DFO, culture results of specimens obtained from bone are more accurate than those from soft tissues, even from deep specimens near the bone [15]. Specimens of bone, compared to those of soft tissue, generally grow fewer isolates, and most often the predominant (and frequently the sole) pathogen is *S. aureus*. A specimen of bone should be obtained by aseptic sampling, either at the time of open surgery or by percutaneous puncture though closed and uninfected skin. Bone specimens taken through an open wound are suboptimal, as they are likely to grow organisms that are contaminants [16]. As with all bacterial cultures, results from patients receiving current antibiotic therapy, including iterative antibiotic treatment related to surgical procedures, may be falsely-negative [12] or yield antibiotic-resistant pathogens that were selected for by this treatment. Indeed, a single-center evaluation in Geneva with 2480 adult patients with orthopedic infections found that the risk of developing a new skin or soft tissue infection (SSTI) was 10% during iterative debridement accompanied by concomitant antibiotic therapy [13]. The microbial etiology of these infections is difficult to predict, as the infections caused by organisms resistant to the currently administered antibiotic agents are already predominant after just the 2nd debridement [13]. This change of pathogens, and their antibiograms, by the selective pressure of antibiotic therapy is particularly common in DFOs occurring in a person with ipsilateral limb ischemia.

### 3.2. General Therapeutic Approaches

Before the 1940s, complete surgical resection was the only successful method of eradicating DFO. There are now many published cases series [17,18], and at least one randomized controlled trial [19], demonstrating that for properly selected patients with DFO, antibiotic therapy without surgery can offer similar results to those with surgery. A review of 10 studies of DFO managed with non-surgical antibiotic treatment found remission rates of 64% to 83% [20]. The most recent and comprehensive guidance for treating DFO is found in the 2019 update of the guidelines on infection from the International Working Group on the Diabetic Foot (IWGDF) [8], which provide rigorously reviewed recommendations on managing all aspects of diabetic foot infections.

### 3.3. Surgical Treatment

Surgical resection of all infected and necrotic bone was the first, and is still a common, approach to treating chronic DFO [21]. Surgery may be required in several situations, such as when: bone protrudes through the ulcer; imaging reveals extensive bone destruction [1]; there is progressive bone damage in a patient undergoing antibiotic treatment; the soft tissue envelope is destroyed; or, there is gangrene or spreading soft tissue infection [22]. The presence of limb ischemia or soft tissue infection (and especially both) in a case of DFO is associated with a worse prognosis for successful treatment. One study reported that when neither of these factors complicated DFO, conservative surgery achieved 100% success, while in the cases with ischemia and spreading soft tissue infection 78% required some type of amputation and the mortality rate was 13% [23]. The planned surgical approach to DFO should balance the benefits and risks of removing as much infected bone as possible against those of preserving viable tissue to aid foot function [22]. In the hands of experienced surgeons, “conservative” (foot sparing) surgery often eradicates infection and produces more acceptable anatomic outcomes [24]. A 2021 narrative review of the literature, which included 14 studies that described seven types of conservative surgical procedures for treating DFO of the forefoot, concluded they were safe and effective, with overall healing rates of 80% to 100% [25]. A minimally invasive distal metatarsal diaphyseal osteotomy (DMDO) procedure has been described as effective for treating complicated DFUs under lateral metatarsal bones, while minimizing various surgery-related complications [26].

#### 3.3.1. Surgery for the Prevention of Future DFO Episodes

The concept of preventive surgery [2,8] for persons with diabetic foot complications is gaining momentum. In the presence of flexible forefoot deformities, such as claw or hammer toes, percutaneous toe flexor tenotomies appear to be effective in reducing soft tissue ulceration, with minimal risk of wound healing complications [27,28,29]. If dorsiflexion of the ankle is limited to less than five degrees (equine deformity) this restricts the leg from rolling over the foot during the late stance phase of walking, thus placing excessive pressure on the plantar forefoot. Older clinical trials demonstrated that Achilles tendon lengthening decreased plantar pressure over the forefoot and reduced recurrence of ulceration of the plantar aspect of the forefoot in patients with limited ankle dorsiflexion. A more recent study by, Kim et al. [30] recommended a plantar fascia release procedure, as it could be associated with fewer complications, and advocated for this technique before considering Achilles tendon lengthening. Gastrocnemius recession (release) is another technique reported to decrease plantar pressure, which thereby helps treat ulcers in the forefoot and midfoot [25]. Clinicians should, however, generally avoid using these surgical procedures in patients with a complex clinical situation.

#### 3.3.2. Surgical Amputations

**Figure 1:**
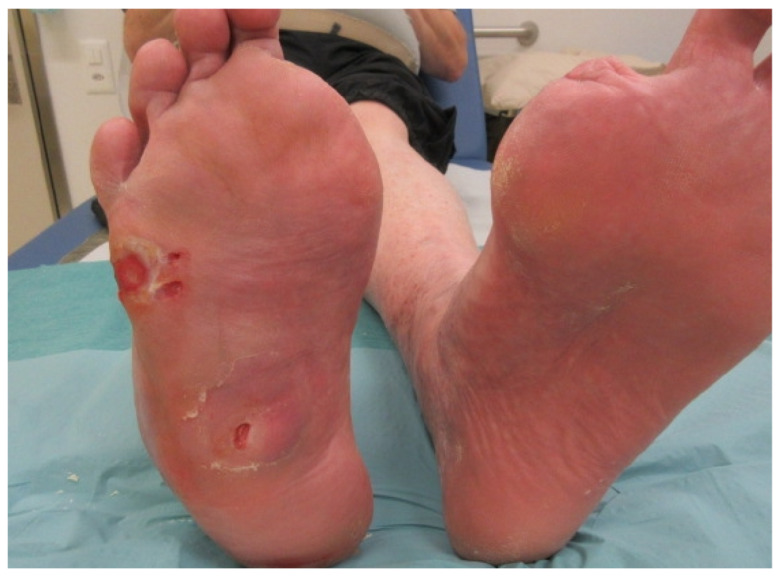
Infected ulceration of the lateral edge of the right foot in a man with diabetic Charcot neuro-osteoarthropathy (with previous amputations of both great toes). Note the collapse of the midfoot, with consequent pressure-related ulcerations, a long-standing clinical problem. The ulcer on the lateral foot recently became infected and was found to have underlying bone involvement. As shown in this photograph, the manifestations of infection in a diabetic foot ulcer may be minimal at the beginning, but can progress rapidly. There is somewhat more pronounced erythema and induration proximal and dorsal to the ulcer. The patient noticed new pain at the site and a sudden change in the color of the foot. He had no fever or visible purulent secretions. This case illustrates that: infection in the diabetic foot is almost always due to underlying problems (such as foot deformity or peripheral neuropathy); even deep infection may present with initially relatively minimal signs and symptoms; clinician’s should consider osteomyelitis in every diabetic patient with a foot ulceration. (Photograph obtained with permission of the patient).

Total amputation of all necrotic and infected tissue is probably the easiest (for the surgeon, if not the patient) and surest way to achieve rapid cure of DFO, at least in the short term [31]. However, this comes at a high price from the point of view of mechanical sequelae, energy expenditure, post-surgical or post-anesthesiology complications, costs and quality of life [32,33]. Moreover, amputation itself, especially in the absence of reversing the reasons for the patient’s initial infection, does not protect against secondary surgical site infections [13], or new DFO episodes [2]. Figure 1 shows an example of a non-corrected, chronic Charcot neuro-osteoarthropathy foot deformity in a patient who continued to have pressure on the lateral foot, with consequent ulceration due to a lack of compliance with the prescribed off-loading. Unfortunately (but not surprisingly), this ultimately led to secondary infection of the soft tissue, then the underlying bone. Currently, most specialists suggest that unless there is severely destroyed bone, amputation should not usually be the primary surgical approach to deal specifically with bone infection [1,5]. Fortunately, there are now many techniques for selective resection, amputation, and reconstruction available to the surgeon to deal with the infected diabetic foot [5].

During preoperative planning for DFO surgery in a patient with lower limb ischemia, surgeons often request transcutaneous oxygen pressure (TCPO_2_) measurements to select the most appropriate amputation level. While 35 mmHg is considered an acceptable threshold for uneventful stump healing [34], this number must be interpreted cautiously and in conjunction with other factors. For example, to explore this issue using modern measurement devices, our Zurich group analyzed 303 lower extremity amputations in 211 patients with previous TCPO_2_ measurements and found that in 26% the stump failed to heal [34]. Using a TCPO_2_ threshold of 35 mmHg did not discriminate well between healing success and stump failure: sensitivity 58%, specificity 48%, positive predictive value 56% and negative predictive value 50%. Furthermore, a TCPO_2_ cutoff level of 20 mmHg yielded the same predictive values as 40 mmHg. By multivariate analyses, there were no significant associations between proximal TCPO_2_ levels and “stump failure”. Finally, receiver-operating-curve and area-under-the-curve analysis ratios were around 50% to 60%, which statistically means quasi-equivalence related to just chance. Of note, available studies in the literature often suggest there is a decisive threshold for the prediction of stump healing, or refer to the auxiliary help of TCPO_2_ measurements, but they fail to provide strict thresholds. Many trials also failed to determine any thresholds, because in everyday practice multiple factors influence stump healing, including infection, surgical techniques, hematoma, and patient compliance. We believe that for foot amputations, the TCPO_2_ level may confirm the clinical impression but does not replace it; surgeons should avoid relying solely on this measurement to select the level of amputation [34].

For the surgeon, amputation involving the forefoot is technically different from that involving the hindfoot, which requires specific experience and expertise. Calcaneal osteomyelitis is an uncommon presentation of DFO (generally comprising <15% of cases) with a somewhat different epidemiology, clinical features, and approach to management. These patients, compared to those with non-calcaneal DFI, more often require special surgical techniques and off-loading approaches [35,36,37]. Undertaking any calcaneal amputation procedure requires that the patient’s posterior tibial artery is patent. If patency is not present or restorable, transtibial amputation is usually indicated. Partial calcanectomy, despite a high clinical failure rate, is most often indicated for calcaneal osteomyelitis, and fortunately has a limited adverse effect on walking ability [37]. When there are no specific restrictions to the choice of surgical procedure, we recommend a partial calcanectomy. This procedure allows the patient to be fitted with an orthopedic shoe after the surgical wound has healed, allowing nearly normal weight-bearing and walking without the compromised energy expenditure that accompanies a below knee amputation. Patients undergoing total calcanectomy, however, usually require a prosthesis that is nearly the height of the type required after below knee amputation [35]. Furthermore, we recommend that the surgeon perform an Achilles tenotomy if the tendon lies within the ulcer area.

**Figure 2:**
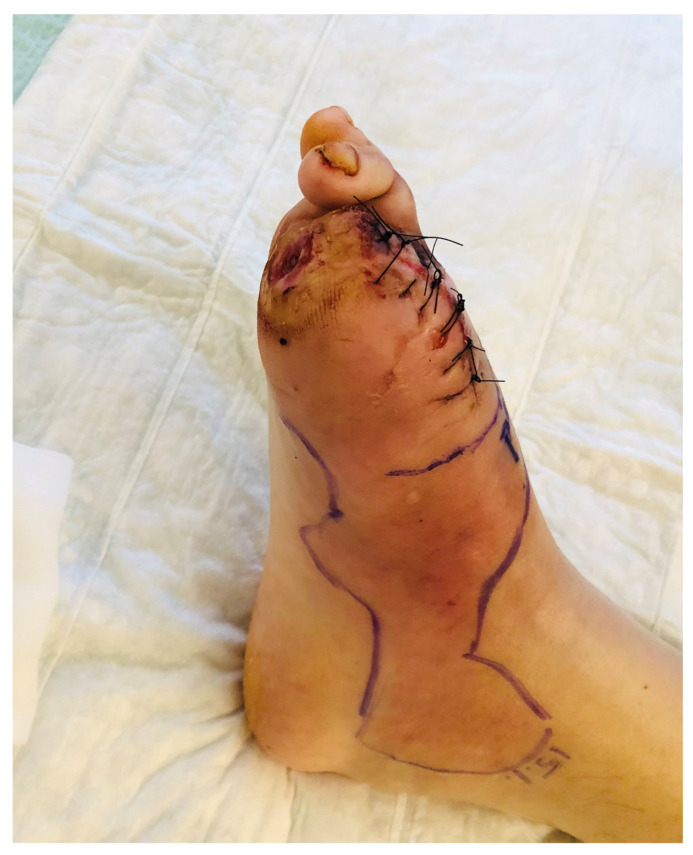
Amputation of the hallux (including the sesamoid bones) in a middle-aged woman for diabetic foot osteomyelitis developing in the setting of a long-standing, neglected plantar ulcer. The infection in the bone was chronic, but was recently complicated by an acute flare of soft tissue infection; this could be a spread from the underlying bone or a new infectious episode caused by a new pathogen. After discussion with the patient, we decided to remove the ulcer along with the underlying infected bone; we debrided the soft tissues, but left the majority of the infected soft tissue, which was treated with systemic antibiotic therapy. In this photograph, taken the first postoperative day, note the substantial residual soft tissue infection, along with a small postoperative hematoma in the forefoot. (Photograph obtained with permission of the patient).

Certainly, some patients with DFO require lower extremity amputation if they have extensive bone destruction, or widespread or difficult to control soft-tissue infection, and especially if they are already non-ambulatory. Other possible indications for amputation may include: ischemic pain; progressive necrosis; severe foot deformities; recurrent foot ulcers; the presence of osteosynthetic material that requires removal; or the patients’ wish to move beyond a conservative approach. Surgeons (and their patients) should, however, be wary of amputation of an acute bone infection that has occurred in the setting of a surgical site infection in the diabetic foot, e.g., after elective surgery for any indication [38]. In many instances, a required amputation does not need to be total, but only sufficient to removed infected tissue unlikely to respond to just antibiotic therapy (Figure 2). This would usually be followed by an appropriate course of antibiotic therapy to eradicate remaining soft tissue or bone infection (see below).

#### 3.3.3. Surgical Reconstruction

One goal of elective DFO surgery is to primarily close the wound in the operating theater, rather than leaving it open for secondary closure. The latter technique, in its most extreme form referred to as a guillotine approach, may be necessary in the presence of limb or life-threating soft tissue infections. Past dogma was to resect as much of the infected soft tissue and bone as possible, with the aim at increasing the likelihood of arresting the infection [39]. More recently, several surgical teams have advocated changing surgical tactics towards sparing as much of the soft tissue and viable bone (even if potentially infected) as possible, a technique with which they have reported good outcomes (Figure 2). Part of this approach relies on the ability to reconstruct the affected foot once the infection has been eradicated. Certainly, for some procedures, such as tissue grafting, ensuring an uninfected surgical field is crucial. One systematic review of 18 studies of lower extremity wounds in diabetic patients identified infection as the main cause for early flap loss [40]. In non-infected flaps, lack of healing was associated with anastomotic failures, local thromboses, stress on the graft edges, or arteriopathy [40]. Hence, the first step in the diabetic foot reconstruction is control of any infection.

### 3.4. Systemic Antibiotics

#### 3.4.1. Antibiotic Stewardship in DFO

In contrast to bacterial infections of sites such as the urinary or lower respiratory tract, infected bone rarely heals in the absence of appropriate anti-infective therapy. Thus, virtually all cases of DFO, whether they undergo any surgical resection or not, require some antimicrobial therapy, usually with systemic antibiotics. But, clinicians must bear in mind that antibiotic-related adverse events are frequent in all types of infections. In randomized-controlled trials involving the diabetic foot, their reported incidence ranges between 15% and 30%, mostly occurring during the first three weeks of therapy [41,42,43]. Thus, in a patient with recurrent diabetic foot problems, clinicians should avoid prescribing antibiotics to treat contaminated superficial wounds, for which there is no proven benefit [41], but only use them in infected wounds, where there is a clear need. On the other hand, clinicians should not always prescribe antibiotics based solely on the academic consideration of the presence of infection, even if contamination is excluded according to international guidance [7,8]. Consequently, it may be appropriate to withhold futile antibiotic therapy in some cases, such as in a patient with complete toe bone destruction who refuses to undergo surgery. For such a patient, it may be appropriate to administer antibiotic therapy for relatively brief periods with the goal of suppressing local worsening of infection.

#### 3.4.2. Route of Antibiotic Administration

Some antibiotics (such as β-lactam agents) do not penetrate well into bone, at least based on the suboptimal methods clinically available to assess this issue. To ensure achieving adequate bone levels of antibiotics, clinicians have therefore long assumed that high serum concentrations are needed [43,44]. Achieving high serum levels to treat DFO was thought to require parenteral (generally intravenous) therapy. For almost forty years, however, evidence from case reports and case series suggested that therapy with orally administered antibiotics that had high bioavailability could successfully treat DFO. Recently, strong evidence supporting this view emerged from the OVIVA study, a randomized, controlled, multicenter trial in the UK that enrolled 1054 evaluable patients who were treated for complex bone and joint infections (including DFO) [45]. This study demonstrated that treatment during the first six weeks with oral antibiotic therapy regimens (after about a week of intravenous therapy) was noninferior to entirely intravenous antibiotic therapy regimens, and it was also associated with fewer intravenous catheter-related complications and lower financial costs [45]. Supporting these findings are data from our recent retrospective cohort analysis from Switzerland assessing the role of oral amoxicillin/clavulanate in treating DFI [46]. We reported on the results of 794 cases, including 339 with DFO, in whom we found no difference in clinical outcomes if they were treated with oral β-lactam antibiotics from the start, or when prescribed only for the second half of the course. The rate of clinical remission in patients treated with this oral β-lactam agent was 74%, similar to that for patients with DFO treated with other antibiotic regimens [46].

#### 3.4.3. The Potential Role of Rifampin in DFO

Rifampi(ci)n is an antibiotic agent with several characteristics that make it potentially attractive for treating osteomyelitis: it is well absorbed when taken orally, has good penetration into bone, and has high activity against the biofilm organisms that often infect bone, including *S. aureus*. Recent interest in the potential value for adding rifampin to combination therapy for DFO led to an observational cohort study using the database of the US Veterans Health Administration [47]. They found that among 6174 patients treated with antibiotics and without surgery for DFO, only 130 (2.1%) received therapy with rifampin. Of note, these rifampin-treated patients had a significantly lower rate of mortality and amputation within two years of diagnosis compared to those treated without rifampin (odds ratio 0.65, *p* = 0.04). Spurred by these findings, this group is currently conducting a randomized controlled trial of six weeks of rifampin therapy (versus placebo) added to conventional treatment (without rifampin) for DFO to see if this adjunctive therapy reduces foot amputations [48].

As part of the work the IWGDF undertook to update the 2019 DFI guidelines, they conducted a systematic review of publications on all types of intervention used for management of DFI [49]. They identified 11 studies specifically conducted in patients identified as having DFO. The authors deemed the quality of most of the studies to be good, and found no significant differences in the outcomes between the various treatment arms, except for poorer outcomes with tigecycline compared to ertapenem. They concluded that the main advantages to treating DFO “medically” (with antibiotics) are to avoid biomechanical changes after surgery, and that it may be more cost effective. Commonly used and evidence based “standard” antibiotic recommendations for treating DFO based on the published literature [7,49,50], including the most recent International Working Group on the Diabetic Foot (IWGDF) guidelines published in 2019 [8], are shown in Table 1.

#### 3.4.4. Duration of Antibiotic Therapy

Because of the difficulty in treating bone infection, recommendations for the duration of antibiotic therapy are generally for a considerably longer duration than for soft tissue infection, typically 4–6 weeks. Many clinicians treat even longer, especially if all necrotic and infected bone has not been resected [2]. The limited published evidence has, however, demonstrated no benefit for administering antibiotic therapy for longer than six weeks. Certainly, prolonged treatment of DFO (as with other infections) is associated with adverse effects. For example, a study from Dallas of 143 patients with biopsy-proven DFO found that 33% developed acute kidney injury [51]. One open-label multicenter, controlled randomized study from France compared DFO cases that were undergoing non-surgical treatment with six-weeks versus twelve-weeks of antibiotic therapy [52]. Among the forty evaluable patients, the remission rate was 65%; there were no significant differences in remission outcomes between the treatment groups, but significantly fewer gastrointestinal adverse events in the six-week group [52].

Similarly, we assessed in a retrospective cohort analysis from Switzerland employing a cluster-controlled Cox regression model, factors related to remission of DFIs, including DFO [31]. We found that DFO episodes treated with <3 weeks of antibiotic therapy had similar outcomes to those receiving >3 weeks. Also, outcomes were not significantly different between episodes treated with more than one week of intravenous therapy than for shorter durations of intravenous therapy. Based on these observations, we reported on a randomized, non-inferiority pilot trial in Geneva that compared clinical remission and adverse event rates in patients with DFO who underwent surgical debridement and were then randomized to either three weeks or six weeks of antibiotic therapy [42]. Among 93 enrolled patients, remission of infection was noted in 84% of patients in the three-week arm compared to 73% in the six-week arm, and the rates of adverse events were similar. The same group of Swiss investigators is currently conducting a larger trial (with a planned enrollment of 400 diabetic patients with soft tissue or bone infection of the foot) to see if they can confirm the results of this pilot study [53]. Based on currently available evidence, we think it is DFO usually does not need to be treated for more than six weeks, and even shorter durations may soon be proven to be sufficient.

Obtaining serial measurements of serum inflammatory markers, such C-reactive protein (CRP), are not helpful in the predicting clinical treatment failure. Among 93 DFO in a prospective observational study we conducted in Geneva, the initial and the final CRP values differed minimally between the groups with remission or failure. Equally, the relative CRP drop (ratio of the final CRP divided by the admission level), as well as the numbers of normalized CRP levels at the end of therapy, were similar for the two groups. Of note is that the clinical impression of the treating physicians was as accurate in predicting outcome as the iterative CRP samplings [54].

#### 3.4.5. Antibiotic Therapy after Amputation for Residual Infection

Patients who undergo surgical resection for DFO often also receive post-operative antibiotic therapy, based on the presumption that there is occult infection in the remaining proximal bone stump. While several research groups have reported finding such residual infection by various microbiological techniques [55], clinically only a small minority of stump complications are due to infections [56]. Prolonging antibiotic therapy after surgical amputation of DFO is usually unnecessary if an experienced surgeon feels confident that all infected bone and soft tissue have been removed [56]. However, in the majority of cases, the surgeon cannot be certain of this based only on the intraoperative appearance. There is no widely accepted standard for clinical practice in these cases; various centers have developed strategies using microbiological assessment of residual bone, systematic empirical continuation of antibiotics, or case-by-case decision [8]. Moreover, methods used to assess residual bone stump infection vary, and include biopsy through a clinically uninfected area with new sterile instruments or open biopsy of the surgical site.

An expert group from France led by Senneville advocates that prescribing 1–3 weeks of additional antibiotic therapy would be enough if all visibly infected bone has been resected [15]. Kowalski et al. from the USA demonstrated that patients with DFI who underwent amputation and had positive bone resection margins for residual osteomyelitis (diagnosed histologically or microbiologically) had more treatment failures and re-amputations than those with negative margins (44% versus 15%) [57]. Atway et al. from the USA reported in a series of diabetic patients a 41% incidence of positive bone resection margins among 27 bone amputations, compared to a 23% incidence among 13 patients following disarticulation [58]. Positive margins suggesting osteomyelitis were associated with worse outcomes, despite a median duration of 25 days of post-surgical antibiotic therapy. In contrast, Rossel et al. from Switzerland reported different results among 239 amputated DFO episodes followed for a median of two years after the index episode [56]. After amputation, the median duration of antibiotic administration was seven days, but in 109 cases (25%), antibiotics were discontinued immediately after surgery. In a multivariate analysis, they found that neither the total duration of postsurgical antibiotic administration nor immediate postoperative antibiotic discontinuation were associated with the failure rate [56]. In a report from Saltoğlu et al. from Turkey, who treated a series of DFI patients with a total excision of infected bone, administering just five days of post-surgical antibiotic therapy was largely sufficient, although their study was not specifically aimed at the question of post-amputation antibiotic continuation [59]. The first interim analysis of an ongoing prospective trial of DFI patients in Zurich, in which the post-amputation antibiotic therapy for residual DFO is randomized to 1 versus 3 weeks [53], shows there are no apparent differences between the groups. Based on the evidence available to date, we encourage clinicians to sample the post-resection residual bone stump and administer prolonged antibiotic therapy to patients whose sample demonstrates evidence of osteomyelitis, especially when they were sampled through the operative site [60]. In addition to limiting this prolonged antibiotic treatment to just those who have evidence of residual infection, this protocol also enhances the likelihood of identifying the true bone pathogens and their current antibiotic susceptibilities [13,53].

#### 3.4.6. Intra-Osseus Local Antimicrobials

For decades clinicians have treated DFO with a variety of local antimicrobial agents (particularly gentamicin, tobramycin, or vancomycin) delivered directly into infected bone using several different methods, including in the form of beads (usually polymethylmethacrylate and more recently calcium sulfate/hydroxyapatite), spacers or cement [1,61]. These agents have been used not only to deliver antibiotics to treat bone infection, but to fill dead space, and in some cases to try to prevent recurrent infection [62]. The agents used for this treatment should ideally: be biocompatible; have minimal toxicity; allow for osteointegration; and, offer prolonged drug release. Although local antibiotic treatments are widely used for DFO, there is little high-quality evidence on the appropriate indications, best techniques, proper dosages, types of antibiotics, elution properties or pharmacokinetics [63]. The most recent published report on this topic is a retrospective review of patients with forefoot DFO who did or did not have perioperative antibiotic-impregnated calcium sulphate implanted [64]. They found that the antibiotic implant did not improve the rate of, or shorten the time to, healing, nor reduce the postoperative amputation rate. It did, however, reduce recurrences of DFO, but at the price of about a third of the patients having wound leakage that lasted for a couple of months [64].

#### 3.4.7. Clinical Pathways, Antibiotic Stewardship and Multimodal Interventions

A way forward to improving treatment of patients with DFO by optimizing available resources includes developing evidence-based clinical pathways, following the recommendations of international guidelines [4,8] and undertaking local or nationwide quality of care projects. The 2019 update of the IWGDF DFI guideline provides an authoritative, international, evidence-based approach to diagnosing and treating DFOs, with 27 recommendations supported by systematic reviews [4,8]. In Switzerland, we have also developed bundled antibiotic stewardship principles specifically for managing DFI [65]. Given how common DFOs are, and how difficult they are to treat, they are probably among the most frequent conditions leading to antibiotic overuse worldwide [65]. Excessive (overly broad-spectrum, or prolonged) antibiotic therapy, while unlikely to lead to better patient outcomes, often results in increased financial costs, drug-related adverse events, and the development of antibiotic resistance. In our review of the limited literature assessing the value of antibiotic stewardship in community and hospital settings, we concluded that the most effective measures were: making a correct infection diagnosis; prescribing an antibiotic regimen with the narrowest effective spectrum; and, limiting the duration of antibiotic treatment. Various administrative approaches, such as having governments establish specialized diabetic foot care centers and providing regular workshops and public educational lectures, could potentially reduce inappropriate antibiotic use [65]. Several adjunctive measures (e.g., hyperbaric oxygen therapy, negative-pressure therapy, direct instillation of antimicrobials) have been used for treating DFO, but none have clearly demonstrated benefit in properly designed clinical trials [49].

To date there is no published evidence that treatment of patients in specialized centers leads to more rational antibiotic therapy. Nevertheless, we believe that following regional or international guidelines can likely help ensure patients are treated with evidence-based therapies underpinned by antimicrobial stewardship principles, thereby helping avoid unnecessary, overly broad-spectrum and unnecessarily prolonged antibiotic treatments for DFIs [65]. However, these guidelines are usually written by infectious diseases specialists and are mostly based on clinical experience and theoretical considerations. Hence, their quality of care approach is limited, because delivering knowledge is not as effective as disseminating advice on adopting proven good practices methods. Despite published advice, many healthcare workers are not sure what to do when faced with complex infections, unhappy patients, inordinate demands for their time and possible wasting of resources. Like other complex problems, optimally managing DFO requires interdisciplinary input from several types of specialists, including non-physicians. Assembling an effective diabetic foot care team, with members given dedicated time and adequate resources, is perhaps the most effective way to help both patients, and the health care providers serving them.

### 3.5. Outcomes of Therapies

Defining successful resolution of DFO is even more difficult than defining its presence. Deeming the outcome of treatment a clinical success usually requires that there be healing of overlying soft tissue infection and wounds, with a return of any abnormally elevated inflammatory markers (especially the erythrocyte sedimentation rate) to normal. Among the few studies that report outcomes of treatment for DFO is a retrospective review of 275 hospitalized patients in the UK, 45% of whom had DFO [66]. DFI cases were not clearly separated by the presence or absence of osteomyelitis, but only 22% of ulcers healed without surgery, and 72% had minor (60%) or major (12%) amputations [66]. A retrospective 2021 study from Costa Rica that reviewed 150 patients who underwent surgery for DFI compared those with DFO to those with only soft tissue infection [67]. Surprisingly, they found no significant differences in length of hospital stay, duration of antibiotic treatment, time to healing, limb salvage or recurrence of infection [67].

Most clinical failures after DFO treatment occur within a few months, or signs and symptoms of infection may even “persist” during ongoing therapy. However, cultures of appropriate specimens (preferably bone) in these clinical recurrences often yield a different bacteriological constellation than the prior episode. This suggests they may actually represent a new episode (reinfection) on a former problematic anatomical site, rather than a relapse of the original infection [68]. Indeed, we found in a study of subsequent episodes of DFI among 482 patients (39% of which were complicated by DFO) followed for a mean of 3.3 years that half had at least one subsequent episode [68]. Among the recurrent DFIs the causative pathogens were different in 57% of cases suggest, but of note the pathogens were usually not more resistant to standard antibiotics used for treatment than were those in the previous episode [68].

It is important to recognize that surgical procedures for DFO can also yield unsatisfactory outcomes. There are no data to support the widely held belief that outcomes are better with surgery than with antibiotic therapy in the long term, because the inherent problems leading to DFO (peripheral neuropathy, foot deformities, improper foot care) are rarely reversed by surgery any more than by antibiotics. As one example of the outcomes with the common procedure of partial toe amputation, a study from Switzerland found that despite professional wound care many needed further surgeries, e.g., proximal re-amputation (39%), of which 11% were major and 25% were minor amputations [69]. Similarly, a retrospective review from the USA found that the risk for re-amputation after partial 1st ray amputation in diabetic patients was 42% after a mean of 34 months follow-up [70]. The authors suggested that surgeons might consider at the initial presentation a more proximal level amputation, such as transmetatarsal, to provide a more functional and reliable residual weightbearing foot. However, a meta-analysis of re-amputations after transmetatarsal amputation found that among 1453 procedures, the major re-amputation rate was 30% [71]. The authors speculated that these findings should raise questions about the conventional wisdom of performing primary transmetatarsal amputation in lieu of other minor amputations, such as partial first ray amputation. On the other hand, it could be that transmetatarsal amputation should be replaced by more proximal levels in order to save time, suffering and resources. Clearly, we need more data to help surgeons and patients make these difficult decisions. In surgical procedures for patients with calcaneal DFO, the failure rates are higher, with one study from Switzerland reporting that after partial calcanectomy 29% of patients needed a secondary amputation [35]. The surgeons from Zurich reporting these results found that among all patients undergoing revision surgery after total calcanectomy, 50% had to undergo secondary amputation, suggesting that this is not the best option in calcaneal osteomyelitis [35].

## 4. Discussion

Studies published in the past few years studies have provided much useful new evidence on optimizing DFO treatment. On the surgical side, it appears that employing more “conservative” (bone sparing) operative procedures is clinically effective and may also reduce post-operative problems [1]. On the antibiotic side, many patients with DFO can be treated with predominantly oral (rather than intravenous) therapy, with similar remission rates, fewer adverse effects and lower financial costs. Furthermore, treating for more than six weeks is not necessary, and as few as 3 weeks might be sufficient [1,42,53]. Finally, in appropriately selected cases, antibiotic therapy without surgical resection can resolve forefoot DFO. Available literature [72] suggests that primarily medical (antibiotic) treatment is most appropriate for infections confined to the forefoot in a patient with good lower extremity arterial perfusion, with no exposed bone or when surgery is not practical or seen favorably by the patient. On the other hand, primarily surgical treatment (usually accompanied by antibiotic therapy) is generally best for patients in whom there is exposed bone or joint, necrotic soft tissue, a fluid collection or abscess, advanced bone destruction or who are at high risk for antibiotic resistant pathogens or antibiotic-related toxicity.

The main limitation of this review is that it is not a formal systematic review or meta-analysis, but rather a less scientifically robust narrative review. Strengths include the fact that we have given a literature based review of a complex topic, supplemented by our combined decades of experience as clinicians and researchers in this field. As noted, there are several ongoing trials that should soon further inform our approach to managing this common and difficult infection. There are research programs looking at new topical antimicrobials, bacteriophages, new systemic antibiotic agents, shorter duration of anti-infective regimens, and maybe more sophisticated off-loading devices and revascularization techniques [2,3]. However, even the best therapeutic interventions cannot prevent new or recurrent episodes as long the major predisposing factors for DFOs persist, i.e., peripheral neuropathy, peripheral arterial disease, patient non-adherence, and foot deformities. Among these factors, perhaps the one most amenable to treatment is improving arterial supply, certainly with revascularization and perhaps (at least partially) with angiogenesis. For example, we have conducted a series of studies with adipose stem cell lines ex vivo [73] and of proangiogenic TIE-2 monocytes from venous blood that suggest we might soon enable neovascularization [74]. Of course, the clinical implication of these laboratory findings on therapeutic neo-angiogenesis will require further studies.

## 5. Conclusions

DFO is a common and growing problem that is difficult to treat. Table 2 offers a basic approach to diabetic person with possible foot osteomyelitis. As we have tried to show in this review, obtaining good outcomes depends upon several key approaches: (1) following published evidence-based guidelines; (2) adhering to the published principles of antimicrobial stewardship and optimal surgical principles, and (3) employing validated diabetic foot care pathways and involving interdisciplinary foot care teams.

Additional key points are that while major amputations were once common for DFO, with improved diagnostic and surgical techniques, it is now frequently possible to perform less ablative procedures, at least for the first therapeutic approach. Also, antibiotic therapy can often be administered predominantly by the oral route, and for shorter durations than have been commonly been used in the past. New ways to use old antibiotics, as well as new antimicrobial agents and approaches make us optimistic about achieving even better outcomes in the next few years.

## Figures and Tables

**Table 1:** Antibiotic agents to consider for treating diabetic foot osteomyelitis based on oral bioavailability and bone concentration (based on references [7,8,49,50]).

Infection Severity	Pathogens	Possible Antibiotics	Comments
*Mild*	*Staphylococcus aureus* (MSSA); *Streptococcus* spp. Methicillin-resistant *S. aureus* (MRSA)	Levofloxacin Amoxicillin-clavulanate Cephalexin Dicloxacillin Clindamycin Doxycycline Trimethoprim/ sulfamethoxazole	QD dosing; substandard for *S. aureus* Relatively broad spectrum & anti-anaerobic Requires QID dosing; inexpensive Narrow-spectrum; QID dosing; inexpensive Covers most (macrolide sensitive) MRSA & anaerobes MRSA, some gram-negatives; QD dosing MRSA, some gram-negatives; undefined against *Streptococcus* species
*Moderate/Severe*	MSSA; *Streptococcus* spp.; *Enterobacteriaceae*; obligate anaerobes MRSA *Pseudomonas aeruginosa* *MRSA, Enterobacteriaceae, P. aeruginosa, anaerobes*	Ertapenem * Ampicillin-sulbactam Imipenem-cilastatin (other carbapenems) Levofloxacin, or ciprofloxacin, with clindamycin Moxifloxacin Ceftriaxone Linezolid * Tigecycline Vancomycin Daptomycin Piperacillin-tazobactam * Vancomycin plus: - Piperacillin-tazobactam, or - Ceftazidime vs. cefepime, or - a carbapenem	QD dosing. Broad-spectrum anti-anaerobic; poor against *Pseudomonas aeruginosa* Relatively broad-spectrum but not for *P. aeruginosa* or other resistant gram-negatives Broad-spectrum; not active for MRSA; consider for proven/suspected ESBL producing pathogens Both oral and parenteral dosage forms suitable. Limited studies of clindamycin for severe *S. aureus* infections; possible anti-toxin effect QD doing. Broad-spectrum, including anaerobes QD dosing (IV or IM); 3rd gen. cephalosporin Oral and IV; adverse effects, drug interactions Broad-spectrum including MRSA; frequent gastrointestinal upset; less effective than others Narrow-spectrum; rising MICs in MRSA isolates QD-dosing; monitor CPK levels TID or QID dosing Very broad spectrum for empiric therapy in severe infections; narrow spectrum when culture & sensitivity results become available

MSSA: Methicillin-sensitive *Staphylococcus aureus*; MRSA: Methicillin-resistant *Staphylococcus aureus*; QD: Once daily; QID: Four times daily; IV: Intravenous; IM: Intramuscular; MICs: Mean inhibitory concentrations; CPK: Creatine phosphokinase; TID: Three times daily; * = Approved by the US Food & Drug Administration for treating diabetic foot infection.

**Table 2:** This is a brief table reminding clinicians of the basics of approaching the diagnosis and treatment of suspected diabetic foot osteomyelitis. Please refer to the text for more detail.

Basic Approach to a Diabetic Person with Possible Foot Osteomyelitis.
**Diagnosis** - *Clinical*: wound size/depth; visible/palpable bone; soft tissue infection; PAD - *Laboratory*: WBC count; erythrocyte sedimentation rate; C-reative protein; procalcitonin - *Imaging*: Plain X-rays; advanced imaging if needed(MRI, radionuclide scans, PET/CT) - *Cultures*: Deep tissue specimens; bone specimen (surgical or transcutaneous) if possible
**Treatment** - **Surgery** - *Urgent* if needed for soft tissue debridement, or pus drainage - *Elective* in most cases if mainly for bone debridement, resection, or amputation - *Preferred primary* approach for patients with: exposed bone or joint; necrotic soft tissue; fluid collection or abscess; advanced bone destruction; need for other surgical repairs; lack of response to antibiotic treatment; high risk for antibiotic resistant pathogens or antibiotic-related toxicity - **Antibiotics** - *Empirical*: Broad-spectrum, or targeted if available culture results, while awaiting results of culture and antibiotic sensitivity tests - *Definitive*: Baseed on: culture and antibiotic sensitivity results; clinical response to empiric therapy; and, antibiotic stewardship principles - *Preferred primary* therapy for patients with: infection confined to the forefoot; adequate limb perfusion; no tissue necrosis; contraindications to, high risk from, or patient preference to avoid, surgery - **Adjunctive**: no treatments of proven benefit

## Data Availability

There are no original data to make available for this review paper.

## Abbreviations

PAD	Peripheral arterial disease
WBC	White blood cell
MRI	Magnetic resonance imaging
PET/CT	Positron emission tomography/computed tomography
